# Fitting Characteristics of N95 Filtering-Facepiece Respirators Used Widely in China

**DOI:** 10.1371/journal.pone.0085299

**Published:** 2014-01-21

**Authors:** Yanyan Yu, Luman Jiang, Ziqing Zhuang, Yuewei Liu, Xinyan Wang, Juyuan Liu, Mingna Yang, Weihong Chen

**Affiliations:** 1 Department of Occupational and Environmental Health, School of Public Health, Tongji Medical College, Huazhong University of Science and Technology, Wuhan, China; 2 Key Laboratory of Environment and Health in Ministry of Education & Ministry of Environmental Protection, and State Key Laboratory of Environmental Health (Incubating), School of Public Health, Tongji Medical College, Huazhong University of Science and Technology, Wuhan, China; 3 National Institute for Occupational Safety and Health, National Personal Protective Technology Laboratory, Pittsburgh, Pennsylvania, United States of America; 4 Guangzhou Occupational Safety & Health Safety Technology Co.,Ltd, Guangzhou, Guangdong, China; The Ohio State University, United States of America

## Abstract

**Background:**

Millions of people rely on N95 filtering facepiece respirators to reduce the risk of airborne particles and prevent them from respiratory infections. However, there are no respirator fit testing and training regulations in China. Meanwhile, no study has been conducted to investigate the fit of various respirators. The objective of this study was to investigate whether people obtained adequate fit when wearing N95 filtering facepiece respirators (FFRs) used widely in China.

**Methods:**

Fifty adult participants selected using the Chinese respirator fit test panel donned 10 common models of N95 FFRs. Fit factors (FF) and inward leakage were measured using the TSI PortaCount Plus. Each subject was tested with three replications for each model. A subject was considered to pass the fit test when at least two of the three FFs were greater than 100. Two models were conducted fit tests before and after training to assess the role of training.

**Results:**

The geometric mean FFs for each model and trained subjects ranged from <10 to 74.0. The fifth percentile FFs for only two individual respirator models were greater than 10 which is the expected level of performance for FFRs. The passing rates for these two models of FFRs were 44.7% and 20.0%. The passing rates were less than 10.0% for the other eight models. There were 27 (54%) participants who passed none of the 10 FFRs. The geometric mean FFs for both models when the subjects received training (49.7 and 74.0) were significantly larger than those when the same group of subjects did not receive any training (29.0 and 30.9) (*P*<0.05).

**Conclusions:**

FFRs used widely in China should be improved according to Chinese facial dimensions. Respirator users could benefit from respirator training and fit testing before using respirators.

## Introduction

A filtering facepiece respirator (FFR) is a device designed to protect the user from inhaling airborne particles and preserve the health of the respiratory tract. They are routinely used by occupational groups. When engineering and administrative controls are not feasible or effective to reduce dust to acceptable levels, respirators become the last defense and the most simple and efficient method to protect workers [1]. Recent reports indicate that more than 128.7 million workers are exposed to industrial dust in China [2], [3]. A similar situation is also observed in the US, where over three million workers are required to wear respirators to protect themselves from hazards on the job [1], [4]. The most widely used respirators are the N95 FFRs. In addition to reduce industrial dust exposure, FFRs are widely used to reduce inhalation of aerosols from volcanic explosions and sandstorms or to prevent respiratory infections, such as influenza, by the general population [5], [6]. For example, the US Occupational Safety and Health Administration approved the use of N95 FFRs for tuberculosis protection in hospitals [7]. In its widespread using, the protective effect of a FRR receives the most attention.

The protective effects of a N95 FFR is mainly depended on the filter penetration and its fit to face. And the later is decided by design basis. The design of FFRs is based on respirator fit test panels which developed from facial anthropometric data collected from various populations. Currently, respirator fit test panels used in China and most other countries were developed by Los Alamos National Laboratory (LANL) in 1978 [8], based on U.S. Air Force anthropometric surveys conducted in 1967 and 1968 [9], [10]. However, the results from a nationwide anthropometric survey of 3000 respirator users across China, conducted in 2006, showed that Chinese civilian adults have shorter face length, smaller nose protrusion, larger face width and longer lip length compared with Americans [11]. Another study conducted by Yang et al. found that Chinese may have shorter and wider facial characteristics than Americans [12]. This raises the question of whether FFRs designed using LANL specifications will fit Chinese adults. A cross-sectional study in South Africa indicated that 86% of the subjects couldn't pass the fit test when wearing a medium-sized disposable P2 particulate respirator designed based on LANL [13]. Another study conducted by Han and his colleagues suggested to develop a well-fitting FFR for Koreans rather than relying on respirators designed using Western facial dimensions [14]. Therefore, the fit characteristics of FFRs for respirator users need further evaluation.

In China, filtration performance testing for FFRs is similar to that in the US and European countries [15]–[17]. However, the testing of respirator leakage between the edge of the respirator and the facial skin is not conducted. Quantitative fit testing, which ensures a respirator fits properly and professional training in FFR donning aren't required in China.

This study was designed to evaluate fitting characteristics of N95 FFRs used widely in China. The objective of this study was to investigate: (1) whether widely used FFRs fit Chinese adults; (2) the consistency of FFRs leakage between fit test and self-feeling of participants, and (3) the possible effect of training in FFR donning on FFR fit tests.

## Methods and Materials

### Subjects

**Figure 1:**
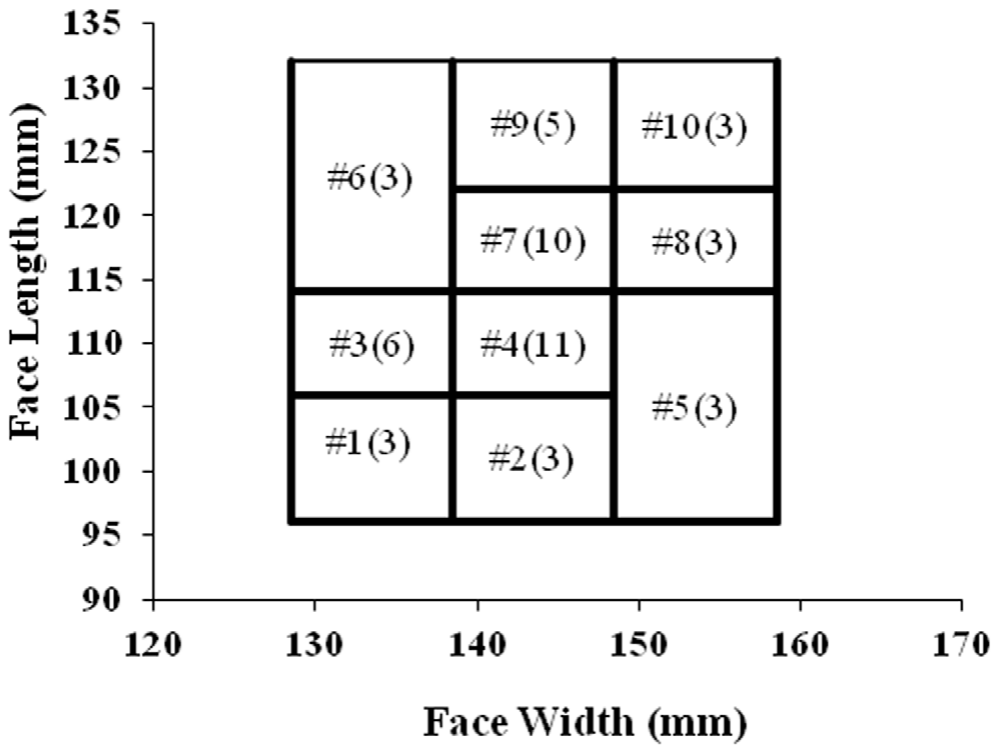
The distribution of 50 participants in Chinese respirator fit test panels. *: Value expressed as the number of participants in corresponding cell.

A total of 50 representative participants (26 males and 24 females, all over 18 years old) were selected from 530 volunteers whose face widths and face lengths were measured to determine inclusion. The Chinese respirator fit test panel based on face width and face length was used to select participants. The number of participants in each cell of fit test panels was representative of the percentage of population in the same cell. At least three subjects were selected for each cell to provide a valid statistical analysis (Figure 1) [18]. The participants could represent the current Chinese civilian workers' facial features [18]. The criteria and requirement for participants also included (1) without respiratory or cardiovascular diseases, (2) no obvious facial deformity, no plastic surgery or facial surgery, (3) scrape beard for a beard before testing [19]. The mean age of the participants was 25.1 years old with a standard deviation of 2.2. Each participant signed informed consent to participate. This study was approved by the Tongji Medical College Institutional Review Board (FWA00007304).

### Respirators

Investigators contacted several major respirator vendors and obtained the sales information for various respirators in the Chinese market. Based on their sales ranking, 10 models of FFRs from four respirator manufacturers (A, B, C and D) in four different countries were chosen in this study. The product features are summarized in Table 1. Samples for each model were purchased in one order to reduce difference among the respirators. Eight respirator models are cup shape and the other two are folding style. Two models have exhalation valves. All these respirator models were certified according to the U.S. National Institute for Occupational Safety and Health, European standards or Chinese standards.

**Table 1:** The base information for 10 models of FFR used in this study.

Manufacturer	Respirator model	Shape	Size	Exhalation valve	Country of manufacturers	Protection level	Approval standard
A	A1	Cup	M	No	China	KN95	GB2626-2006^a^
A	A2	Cup	M	Yes	China	KN95	GB2626-2006
B	B1	Cup	M	No	France	N95	EN149: 2001^b^
B	B2	Cup	M	Yes	France	N95	EN149: 2001
C	C1	Cup	M	No	U.K.	N95	GB2626-2006
C	C2	Folding	M	No	U.K.	N95	GB2626-2006
C	C3	Cup	S	No	U.K.	N95	NIOSH N95^c^
C	C4	Cup	M	No	U.K.	N95	NIOSH N95
D	D1	Cup	M	No	U.S.	N95	NIOSH N95
D	D2	Folding	M	No	U.S.	N95	NIOSH N95, AS/NZS 1716^d^

Abbreviation: FFR, Filtering facepiece respirator.

a GB2626-2006: Respiratory protective — Non-powered air-purifying particle respirator.. Chinese standard);

b EN 149:2001: Respiratory protective devices — Filtering half masks to protect against particles — Requirements, testing, marking (European standard),

c NIOSH N95: 42 CFR 84 Subparts K — Non-Powered Air Purifying Particulate Respirator. (American standard);

d AS/NZS 1716: Respiratory Protective Devices (Australian/New Zealand Standard).

### Inward Leakage (IL) Measurement

Quantitative fit tests were conducted using the TSI Portacount (model 8020) to measure IL following the procedure defined in OSHA's respirator regulation 29 CFR 1910.134 [19]. The tests were conducted in a routine laboratory environment and no supplemental aerosols were used. All participants who were smokers refrained from smoking for at least 30 minutes before the test. A respirator was donned and worn at least 3 minutes prior to testing. Once a participant believed that the FFR was donned properly, fit testing began. Each participant performed the test exercises in the following manner: normal breathing, deep breathing, turning head side to side, moving head up and down, talking, grimacing, bending over, and normal breathing. All exercises last 86 seconds except grimacing exercise which last 22 seconds. When one fit test was completed, the subject removed the respirator and gave it to the test operator. The test operator regulated the respirator to its original configuration (e.g., loosening head straps, straightening the nose-clip) and gave it back to the participant for testing again. The fit tests for each FFR were conducted 3 times. Participants were given a 3 minute adaptation period before each fit test and at least 3 minutes to rest after each fit test [20]. The order of 10 testing FFR models were randomized for each participant [20]. After fit testing, the participants were asked to report their feeling of the FFR leak.

With an aim to evaluate the role of training for respirator donning, this study selected two models of the 10 FFRs (D1, D2) and conducted fit tests before and after the training. For the testing without training (D1F, D2F), all 50 subjects donned the FFR and conducted fit test after reading the respirator manufacturer's instructions themselves, including performing and passing a use-seal check [21]. For the testing after training, the researchers instructed the same 50 subjects to don and doff a respirator and to conduct the use-seal check. The researchers also check if the respirator was worn properly. For the rest of 8 FFRs, all fit tests were conducted after subjects were properly trained.

For all 10 respirators and after properly training, subjects were asked if they could feel any leakage around the face seal area. Their answers (either yes or no) were recorded.

### Filter Penetration and Fit Factor (FF) Measurement

**Figure 2:**
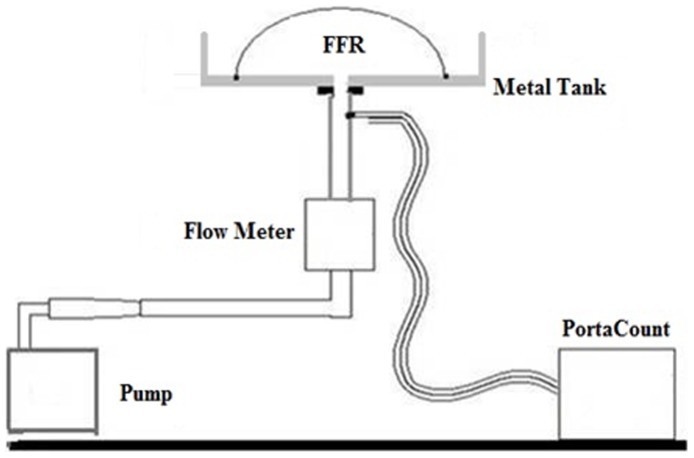
The schematic diagram of filter penetration measure equipments.

Filter penetration was measured for each FFR by TSI Portacount Plus. The edge of the FFR was sealed in a metal tank which was connected to an air pump and the Portacount Plus (Figure 2). Filter penetration was measured while air pump ran at a rate of 30 L/min (simulate the gas flow during labor). Filter penetration was measured three times for each respirator.

The face seal leakage is calculated by inward leakage (IL) minus filter penetration as follows:
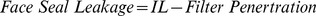



The reciprocal of face seal leakage is a fit factor (FF). The quantitative fit test of one FFR is considered to have passed when FF is equal to or greater than 100. Passing rate is the number of tests that passed divided by the total number of tests (50×3 = 150). A participant is considered to have passed the fit test if at least two of the three FFs are equal to or greater than 100. And passing rate for participant is the number of subject passed fit test divided by the 50 participants.

### Measurement of Head and Face Dimensions

Once a participant was included in the study based on face width and face length, additional 19 head-and-face anthropometric dimensions and height, weight, waist circumference and hip circumference were measured using traditional methods. Head-and-face dimensions included head circumference, bitragion coronal arc, bitragion frontal arc, bitragionsubnasale arc, bitragion chin arc, neck circumference, head breadth, head length, minimum frontal breadth, maximum frontal breadth, face width, bitragion breadth, nasal root breadth, nose breadth, lip length, subnasalesellion length, face length, nose protrusion, selliontragion length, interpupillary distance, and mentonsubnasale [11]. Measurements were determined by means of a sliding caliper and spreading caliper [22].To assure the measuring accuracy, face length and face width of the 50 subjects were measured twice. There was no significant difference between the two measurements.

### Statistical Analyses

Kappa statistics were employed to examine the consistency between fit test and self feeling on leakage of the respirators. The statistic is expressed as: 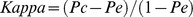 where Pc is the crude agreement and Pe is the expected agreement. The kappa value was interpreted as follows: <0.40 is poor, 0.41 to 0.60 is moderate and >0.60 is good in consistency.

The data for IL and FF were log-transformed because they are log-normally distributed [20]. The geometric mean FFs were calculated for each respirator model and each cell of the fit test panel respectively [23]. The difference between training and no training and among the 10 FFRs was also determined by the independent variables one-way ANOVA. All statistical analyses were conducted using SAS version 9.3 (SAS Institute, Cary, NC).

## Results

### Head-and-face Anthropometric Dimensions for Participants

The mean and standard deviation for head-and-face anthropometric dimensions by gender and all subjects combined is summarized in Table 2. The male subjects had face widths ranging from 135 mm to 158 mm and face lengths ranging from 106 mm to 127 mm. The female subjects had face widths ranging from 130 mm to 148 mm and face lengths ranging from 103 mm to 121 mm. These individuals could represent Chinese adults because the average anthropometric dimensions for 50 participants in this study are similar as those for 3000 Chinese in a previous study [11].

**Table 2:** The mean and standard deviation for all 25 anthropometric dimensions based on gender and all participants.

Dimension	Male(N = 26)	Female(N = 24)	All(N = 50)
Height(m)	1.725±0.065	1.597±0.037	1.663±0.084
Weight(kg)	61.9±8.0	49.4±4.8	55.9±9.1
Waist circumference(mm)	762.5±69.0	677.8±47.6	721.8±72.9
Hip circumference(mm)	935.8±49.2	897.9±45.9	917.6±50.9
Head circumference(mm)	564.2±13.3	546.4±10.8	555.7±15.1
Bitragion coronal arc(mm)	364.4±11.8	353.6±13.5	359.2±13.6
Bitragion frontal arc(mm)	315.3±9.3	296.2±10.2	306.1±13.6
Bitragionsubnasale arc(mm)	302.8±10.8	284.4±10.6	294.0±14.1
Bitragion chin arc(mm)	324.3±13.3	302.5±13.7	313.8±17.3
Neck circumference(mm)	350.2±19.3	293.5±24.5	323.0±35.9
Head breadth(mm)	158.2±6.0	154.0±6.2	156.2±6.4
Head length(mm)	185.9±6.2	175.5±5.4	180.9±7.8
Minimum frontal breadth(mm)	107.5±5.6	105.9±4.4	106.8±5.1
Maximum frontal breadth(mm)	118.4±5.3	114.0±5.2	116.3±5.7
Face width(mm)	146.1±5.8	138.9±5.2	142.7±6.5
Bitragion breadth(mm)	112.4±5.6	104.0±7.3	108.4±7.7
Nasal root breadth(mm)	18.8±1.9	18.7±1.9	18.7±1.9
Nose breadth(mm)	38.4±2.7	35.3±2.1	36.9±2.9
Lip Length(mm)	50.2±4.0	46.9±2.3	48.6±3.6
Subnasalesellion length(mm)	51.4±2.4	47.0±3.4	49.3±3.7
Face length(mm)	118.2±6.5	110.9±5.2	114.7±6.9
Nose protrusion(mm)	19.7±2.4	18.3±2.3	19.0±2.5
Selliontragion length(mm)	71.5±3.8	70.0±3.9	70.8±3.9
Interpupillary distance(mm)	64.1±3.3	61.7±2.0	62.9±3.0
Mentonsubnasalelength(mm)	70.2±4.8	66.8±5.6	68.5±5.4

### Effect of Training on Respirator Fit

As shown in Table 3, geometric mean FFs of the trained group were approximately twice as large as those of the untrained group for both models of respirators. The passing rates for two models in trained group were also much higher than those in untrained group. The differences of geometric mean FFs between the untrained and trained group for two models were significant (*P*<0.05). So were the passing rate and passing rate of participants by chi-square analysis for D1 (*P*<0.05). The results indicated that formal training for respirator donning helped to improve FF.

**Table 3:** Geometric mean fit factor and passing rate for two FFR models when subjects received no training and when the same subjects received proper training.

Gourp and Respirator model	No. of participants	No. of testing	Geometric mean FF*	GSD	Fifth percentile FF	No. of participants pass the testing	Passing rate of participants (%)	No. of respirator pass the testing	Passing rate of respirator (%)
Untrained D1	50	150	30.9^C^	3.4	4.2	9	18.0	29	19.3
Trained D1	50	150	74.0^A^	2.7	14.6	21	42.0	67	44.7
Untrained D2	50	150	29.0^C^	3.2	4.2	6	12.0	24	16.0
Trained D2	50	150	49.7^B^	2.4	11.4	10	20.0	30	20.0

Abbreviation: FFR, Filtering facepiece respirator; FF, fit factor; GSD, geometric standard deviation,

* The geometric mean fit factors with the different superscript letters (A, B or C) are significantly different.

### Inward Leakage (IL) and Filter Penetration

The IL and filter penetration for each model of respirator is summarized in Table 4. The values of filter penetration for 10 models were below 0.1% with the exception of model C2 and D2. The geometric mean ILs of model C1, C2, C3 and C4 were higher than 10% and indicated increased leak. The 10% of IL is a expected value for a FFR and suggests an acceptable level of protection. The 95th percentiles of IL for 10 models ranged from 6.8% to 53.3% and only for model D1 and D2 were less than 10%. The 95th percentile of IL for 10 models of FFR combined was 42.2%. This is more than four times of 10%. These results indicated that ILs of 10 models was high and the participants in this study couldn't expect to achieve the desired level of respiratory protection.

**Table 4:** Inward Leakage (IL) and filter penetration for 10 models of FFRs in this study.

Respirator model	No. of testing	Geometric mean IL (%)	GSD (%)	95^th^ percentile IL (%)	Mean filter penetration (%)	SD (%)
A1	150	4.8	2.1	16.1	0.006	0.004
A2	150	7.0	2.3	27.0	0.066	0.050
B1	150	7.0	2.8	38.1	0.003	0.005
B2	150	7.2	2.1	24.6	0.009	0.004
C1	150	12.2	2.1	39.8	0.002	0.000
C2	150	11.0	2.4	44.9	1.119	0.513
C3	150	14.4	1.9	41.4	0.004	0.001
C4	150	17.7	2.0	53.3	0.013	0.010
D1	150	1.4	2.7	6.8	0.005	0.001
D2	150	2.2	2.3	8.5	0.137	0.132
Total	1500	6.7	3.1	42.2	0.136	0.364

Abbreviation: IL, Inward leakage; FFR, Filtering facepiece respirator; GSD, geometric standard deviation, SD, standard deviation.

### Fit Factor (FF) and Passing Rate

The results of FF and passing rate of 10 models of FFR are summarized in Table 5. The geometric mean FF for 10 models was 15.4. There were significant differences of geometric mean FFs among 10 models of FFR (*P*<0.05). The geometric mean FFs of respirator model D1 (74.0) and D2 (49.7) were significantly higher than those of other respirator models (*P*<0.05). The geometric mean FF of C4 (5.7) was significantly lower than those of other models (*P*<0.05). The fifth percentile FF was 2.4 (range from 1.9 to 14.6) for all the respirators. The fifth percentile FFs for two respirator models (D1 and D2) were greater than 10 which is the expected level of performance for FFR. Therefore, eight of ten respirators used widely in China did not fit the participants very well.

**Table 5:** The geometric mean FFs and passing rate for 10 models of FFR.

Respirator model	No. of testing	No. of participants	Geometric mean FF*	GSD	Fifth percentile FF	No. of participants pass the testing	Passing rate of participants (%)	No. of respirator pass the testing	Passing rate of respirator (%)
A1	150	50	20.9 ^C^	2.1	6.2	2	4.0	6	4.0
A2	150	50	14.6 ^D^	2.3	3.7	2	4.0	4	2.7
B1	150	50	14.3 ^D^	2.8	2.6	2	4.0	6	4.0
B2	150	50	13.9 ^D^	2.1	4.1	0	0.0	1	0.7
C1	150	50	8.2 ^F^	2.1	2.5	0	0.0	0	0.0
C2	150	50	10.5 ^E^	2.6	2.1	1	2.0	3	2.0
C3	150	50	6.9 ^F^	1.9	2.4	0	0.0	0	0.0
C4	150	50	5.7^G^	2.0	1.9	0	0.0	0	0.0
D1	150	50	74.0 ^A^	2.7	14.6	21	42.0	67	44.7
D2	150	50	49.7 ^B^	2.4	11.4	10	20.0	30	20.0
All	1500	500	15.4	3.1	2.4	38	7.6	117	7.8

Abbreviation : FF, fit factor; FFR, Filtering facepiece respirator; GSD, geometric standard deviation.

* The geometric mean fit factors with the different superscript letters (A to G) are significantly different.

Overall, the passing rates of quantitative fit test for all 10 models of FFR were 7.8% (Table 5). The highest passing rate was 44.7% for model D1. The passing rates for model C1, C3, C4 were 0%. There were 23 of the 50 participants who passed the fit test for at least one model. There were 27 (54%) subjects who passed none of the 10 models and could not find an appropriate respirator in 10 models.

### Fit factor information by each cell of the Chinese new fit test panel

Table 6 summarizes the geometric mean FFs of the 10 models of FFR for each cell of Chinese respirator fit test panel. The geometric mean FFs in cell #4 (40.2) was highest among all 10 cells. And lower geometric mean FFs were 15.5 for cell #10, 18.0 for #3 and 19.0 for #9. The results indicated that adults with face dimension in Cell #3, #9 and #10 will be a little difficult to find appropriate respirator among 10 models in this study.

**Table 6:** The geometric mean FFs* and their geometric standard deviation for 10 models of FFRs for cells in Chinese respirator fit test panels.

Respirator model	No. of cell*									
	#1	#2	#3	#4	#5	#6	#7	#8	#9	#10
A1	22.0	13.0	16.8	33.1	37.9	27.8	15.4	30.6	13.8	14.2
	(1.5)	(2.5)	(1.7)	(2.1)	(2.7)	(1.4)	(1.8)	(1.4)	(1.7)	(2.2)
A2	19.7	9.7	10.2	21.5	20.6	17.5	12.0	16.7	13.1	9.2
	(2.0)	(1.7)	(2.0)	(2.5)	(4.6)	(1.7)	(2.3)	(1.8)	(1.5)	(2.1)
B1	9.3	14.7	13.8	17.7	40.0	13.1	16.1	9.8	13.4	4.7
	(1.6)	(3.1)	(3.4)	(3.3)	(1.8)	(2.1)	(2.4)	(2.1)	(2.2)	(2.4)
B2	11.0	11.7	20.9	20.2	16.4	15.3	13.5	10.7	7.7	6.8
	(2.4)	(1.4)	(1.9)	(2.4)	(1.9)	(1.5)	(1.8)	(2.1)	(1.8)	(1.8)
C1	8.9	10.3	13.7	9.1	13.0	8.3	6.0	6.3	6.2	5.1
	(1.9)	(1.3)	(1.4)	(2.3)	(1.6)	(2.0)	(2.0)	(2.2)	(2.0)	(1.9)
C2	8.2	9.2	8.9	16.0	20.1	6.6	9.3	8.4	13.2	4.8
	(1.9)	(2.3)	(2.4)	(3.9)	(2.0)	(1.8)	(1.9)	(3.7)	(1.7)	(2.0)
C3	9.0	8.0	8.7	9.4	7.2	8.8	5.6	4.3	4.8	4.6
	(2.1)	(1.4)	(1.5)	(2.0)	(2.3)	(1.5)	(1.7)	(2.2)	(1.6)	(1.6)
C4	10.2	6.0	5.1	8.3	4.2	7.3	4.3	4.2	5.0	3.8
	(1.8)	(2.0)	(1.8)	(2.3)	(1.8)	(1.8)	(1.7)	(1.4)	(1.5)	(1.4)
D1	147.1	96.4	39.0	72.0	154.6	72.2	74.6	82.1	65.1	61.2
	(1.5)	(2.6)	(5.0)	(3.1)	(2.3)	(1.6)	(1.9)	(1.4)	(2.3)	(2.0)
D2	45.6	47.3	43.2	52.3	87.8	58.1	46.7	48.0	48.0	40.4
	(1.7)	(2.3)	(3.6)	(3.0)	(3.4)	(1.6)	(1.9)	(1.7)	(1.6)	(2.7)

Abbreviation : FF, fit factor; FFR, Filtering facepiece respirator.

* value expressed as geometric mean (geometric deviation).

There are two shapes of 10 models of FFR, eight of them are cup and two of them are folding style. Model D1 and D2 came from the same manufacture. Model D1 was cup respirator and D2 was folding respirator. The geometric mean FFs of model D1 were higher than those of model D2 (*P*<0.01). We also noted that the geometric mean FFs of model D1 for males was larger than those for females (*P*<0.05). On the contrary, the geometric mean FFs and passing rates of model D2 for males were smaller than those for females (*P*<0.05). The results indicated that the cup respirators fit better for males and the folding respirators fit better for females, the same as our previous study [24].

### The consistency of FFRs leakage between fit test and self-feeling of participants

The results of leakage determined by fit test and by self-feeling of participants for 10 models of FFR are summarized in Table 7. Passing was defined as at least two of the three FFs are greater than 100 for fit test and no leakage for self-feeling. There were 219 (43.8%) from total 500 subject-respirators combinations were thought to be passed by the participants. However, only 24 (4.8%) in those 219 subject-respirators combinations were found to have passed by fit test. The results indicated that 39.0% of the time, participants couldn't detect the leakage of the respirators. The consistency of passing rate between fit test and self-feeling are calculated by Kappa coefficients. All of the kappa coefficients were lower than 0.4 (−0.145–0.109) and the total kappa value was 0.066. The results indicated that the consistency between the results of fit test and self-feelings for participants was bad. The participants can't provide reliable feelings of leakage on the respirators.

**Table 7:** Leakage for 10 models of FFR detected by fit test and self-feeling of participants.

Respirator	Self-feeling	Fit test		Kappa value	*P*
		*Pass	Not pass		
A1	Pass	2	19	0.109	0.09
	Not pass	0	29		
A2	Pass	2	20	0.101	0.1
	Not pass	0	28		
B1	Pass	0	12	−0.074	0.42
	Not pass	2	36		
B2	Pass	0	21	0	1
	Not pass	0	29		
C1	Pass	0	22	0	1
	Not pass	0	28		
C2	Pass	1	19	0.059	0.22
	Not pass	0	30		
C3	Pass	0	18	0	1
	Not pass	0	32		
C4	Pass	0	16	0	1
	Not pass	0	34		
D1	Pass	14	24	−0.145	0.19
	Not pass	7	5		
D2	Pass	5	24	−0.058	0.57
	Not pass	5	16		
Total	Pass	24	195	0.066	0.01
	Not pass	14	267		

Abbreviation : FFR, Filtering facepiece respirator.

* Pass was defined as at least two of the three FFs are greater than 100 for fit test and no leakage for self-feeling.

## Discussion

The fit factors (FFs) of 10 widely used N95 FFRs had poor fitting characteristics for participants in this test. The geometric mean FFs of all respirators were 15.4 with range from 5.7 to 74.0. The passing rates (0% to 44.7%) of quantitative fit test for all 10 models of FFR were 7.8% and all were below 50%. Two models of D1 and D2 fit better than other respirators but their geometric FFs were still less than 100. The results showed that 27 of 50 participants could not find appropriate FFRs among these 10 common models in the market. This study indicated that widely used N95 FFRs in China didn't fit well and can't provide desired protection for respirator users.

We used Chinese respirator fit test panel representing current Chinese civilian adults to select the participants for this study. The differences of average anthropometric dimensions for 50 participants in this study and those for 3000 Chinese in a previous study [11] were minimal. That means the participants in this study could represent Chinese adults well. Mean face width and face length for American males were reported to be 143.5 mm and 122.7 mm, and 135.1 mm and 113.4 mm for females [22]. The face width (146.1 mm for male and 138.9 mm for female) of this study were wider than American, and the face length (118.2 mm for male and 110.9 mm for female) for Chinese adults were shorter than American [12], [22], [25]. Face length and face width are main indexes used for defining the panel for half-facepiece respirators [26]. Although difference of face width and face length exists between American and Chinese, FFRs in Chinese market were designed according to US LANL panel. And our previous study found that LANL panel could not cover 20.9% of Chinese adults [11]. The current study confirmed the role of respirator fit test panel in FFR design because we observed that 54% participants could not find appropriate FFRs designed by LANL. Inappropriate panel might be the major reason for low FFs of 10 models of FFR in this study. Similar results were reported by Spies and his colleagues in South Africans [13]. They found that 86% subjects couldn't pass the fit test when wearing a medium-sized respirator which designed based on LANL panel, although 29 volunteers in their study were randomly recruited.

The second reason for low FFs of 10 models of FFR is leakage. Filter penetrations for most respirators were below 0.1% and its contribution to IL was small. Thus, face seal leakage is a major factor which contributes to IL. And high value of IL directly induces low FF for a respirator. The participants felt that most respirators had a serious leak around the edge of FFRs, especially at the nasal and chin region. Similarly, Oestenstad et al. observed that about 89% of all observed leaks occurred at the nose or chin or were multiple leaks [27]. Han and his colleagues also found that the main route leakage for FFRs may not be the filter medium but the face seal [28].

Another reason for low FFs of 10 models of FFR might attribute to the size of respirator. The most of FFRs in the market only have one size, medium. It is well known that one universal respirator is difficult to fit all users [29], even if the quality of the material is very good.

The results of this study indicated that self-feeling for leakage of the N95 FFRs was not reliable. About 39.0% of the time, users with self-feeling of no leakage can't pass fit test. For this reason, we suggest conducting fit testing before selecting N95 FFRs. On the other hand, training in FFRs donning has greatly improved the FF in this study. U.S. federal regulations require the employers to provide fit testing and effective training to employees so they can understand the need, use, limitations, and capabilities of the respirators they wear [19]. However, little professional training in respirator donning is conducted in China and other countries [13], to the best of our knowledge. Thus, the results from this study suggest that government agencies or occupational safety and health organizations should propose a measure to strengthen the training in FFR donning. It is recommended to develop or complete current standard of selection and using respiratory protective equipment [30].

These findings have important public health implications. N95 FFRs are trusted and very commonly used in China and other countries. The users believe they will get enough protection by N95 FFRs when they enter working environment with industrial dusts or infectious aerosols. However, if the passing rates of quantitative fit test for FFRs widely used in China are below 50%, we have to worry about the protective effect of N95 FFRs. These results underscore an urgent need to establish regulations on conducting the qualitative or quantitative fit testing when users choose FFRs and training for FFRs donning.

## Conclusions

This study found that the fit of widely used N95 FFRs on Chinese workers was poor. The respirators used in China regardless of the country of manufacturer, could benefit from considering Chinese facial dimensions in the design process. Presently, respirator users in China could benefit from respirator training and fit testing before using a respirator.
